# The Role of the Built-In Electric Field in Recombination Processes of GaN/AlGaN Quantum Wells: Temperature- and Pressure-Dependent Study of Polar and Non-Polar Structures

**DOI:** 10.3390/ma15082756

**Published:** 2022-04-08

**Authors:** Kamil Koronski, Krzysztof P. Korona, Serhii Kryvyi, Aleksandra Wierzbicka, Kamil Sobczak, Stanislaw Krukowski, Pawel Strak, Eva Monroy, Agata Kaminska

**Affiliations:** 1 Institute of Physics, Polish Academy of Sciences, Aleja Lotnikow 32/46, 02-668 Warsaw, Poland; koronski@ifpan.edu.pl (K.K.); kryvyi@ifpan.edu.pl (S.K.); wierzbicka@ifpan.edu.pl (A.W.); 2 Faculty of Physics, University of Warsaw, Pasteura 5, 02-093 Warsaw, Poland; krzysztof.korona@fuw.edu.pl; 3 Biological and Chemical Research Centre, Faculty of Chemistry, University of Warsaw, Zwirki i Wigury 101, 02-089 Warsaw, Poland; ksobczak@cnbc.uw.edu.pl; 4 Institute of High Pressure Physics, Polish Academy of Sciences, Sokolowska 29/37, 01-142 Warsaw, Poland; stach@unipress.waw.pl (S.K.); strak@unipress.waw.pl (P.S.); 5 CEA-IRIG-DEPHY-PHELIQS, University Grenoble-Alpes, 17 Av. des Martyrs, 38000 Grenoble, France; eva.monroy@cea.fr; 6 Faculty of Mathematics and Natural Sciences, School of Exact Sciences, Cardinal Stefan Wyszynski University, Dewajtis 5, 01-815 Warsaw, Poland

**Keywords:** multi-quantum wells, nitrides, electric field, time-resolved photoluminescence, high-pressure spectroscopy

## Abstract

In this paper, we present a comparative analysis of the optical properties of non-polar and polar GaN/AlGaN multi-quantum well (MQW) structures by time-resolved photoluminescence (TRPL) and pressure-dependent studies. The lack of internal electric fields across the non-polar structures results in an improved electron and hole wavefunction overlap with respect to the polar structures. Therefore, the radiative recombination presents shorter decay times, independent of the well width. On the contrary, the presence of electric fields in the polar structures reduces the emission energy and the wavefunction overlap, which leads to a strong decrease in the recombination rate when increasing the well width. Taking into account the different energy dependences of radiative recombination in non-polar and polar structures of the same geometry, and assuming that non-radiative processes are energy independent, we attempted to explain the ‘S-shape’ behavior of the PL energy observed in polar GaN/AlGaN QWs, and its absence in non-polar structures. This approach has been applied previously to InGaN/GaN structures, showing that the interplay of radiative and non-radiative recombination processes can justify the ‘S-shape’ in polar InGaN/GaN MQWs. Our results show that the differences in the energy dependences of radiative and non-radiative recombination processes cannot explain the ‘S-shape’ behavior by itself, and localization effects due to the QW width fluctuation are also important. Additionally, the influence of the electric field on the pressure behavior of the investigated structures was studied, revealing different pressure dependences of the PL energy in non-polar and polar MQWs. Non-polar MQWs generally follow the pressure dependence of the GaN bandgap. In contrast, the pressure coefficients of the PL energy in polar QWs are highly reduced with respect to those of the bulk GaN, which is due to the hydrostatic-pressure-induced increase in the piezoelectric field in quantum structures and the nonlinear behavior of the piezoelectric constant.

## 1. Introduction

GaN/AlGaN quantum structures are intensively studied because of a wide variety of applications (e.g., in ultraviolet (UV) light-emitting diodes (LEDs) [1,2,3,4,5], photodetectors [6], high-electron mobility transistors [7], and solar cells [8]). GaN/AlGaN multi-quantum-wells (MQWs) grown on polar or non-polar GaN have also been investigated as promising materials for new intersubband devices [9,10].

The early success of violet and blue LEDs was possible because of the fortunate neutralization of dislocation-mediated non-radiative recombination. According to the model proposed by Chichibu et al. [11,12,13], efficient radiative recombination was attributed to the localization of the carriers in In-rich domains, which caused potential fluctuations within the InGaN quantum wells (QWs). This effect has led to the development of blue LEDs with efficiencies higher than 90%. In contrast, much trouble was encountered when trying to extend this technology towards longer (green-yellow) or shorter (UV) wavelengths. Looking at longer wavelengths (green, yellow), the effect of the carrier localization that was beneficial in layers with low indium contents (below 25%) appears to be detrimental in layers with higher In contents (e.g., emission wavelengths λ > 500 nm can be obtained when the In content in InGaN exceeds 30%). The model of the potential fluctuations that are caused by indium segregation also explains the existence of the Stokes shift (i.e., the energetic difference between the absorption edge and the photoluminescence (PL) peak energy), which is frequently observed in the optical measurements of such structures [14]. The energetic position of the absorption edge is defined by the average value of the energy gap (average indium content), whereas the PL occurs because of radiative transitions between the local conduction band minima and the valence band maxima, and so it arises from the regions where the bandgap is lower, and the local indium concentration is larger. The Stokes shift value is, then, an indicator of the degree of localization. 

The Stokes shift can also be influenced by the variation in the temperature. By analyzing the dependence of the absorption and the emission energy on the temperature, it is assumed that the temperature dependence of the absorption edge in a disordered alloy essentially reflects the behavior of the bandgap, so that a regular decrease in the bandgap energy with increasing temperature can be expected, according to the Varshni formula [15] or the Viña model, based on Bose–Einstein statistics [16]. In the case of the PL energy, however, the situation can be different, and the observed behavior of the PL energy of the alloy as a function of temperature may consist of three regions: (i) A low-temperature region, where the PL energy decreases with increasing temperature; (ii) An intermediate-temperature region, where the PL energy increases with increasing temperature; and (iii) A high-temperature region, where the PL energy again decreases with increasing temperature. The observation of such behavior, which is usually referred to as ‘S-shape’ behavior, has been extensively studied and reported in the literature for InGaN alloys and InGaN/GaN QWs, and was explained by thermally induced changes in the degree of carrier localization that were caused by potential fluctuations that were due to indium clustering [17,18,19,20,21,22,23]. Interestingly, ‘S-shape’ behavior has been also reported for AlGaN alloys with low Al contents [24,25] and for GaN/AlGaN heterostructures [26], where the localization cannot be due to alloy inhomogeneities in the wells, since they consist of binary GaN.

Potential fluctuations in GaN/AlGaN QWs are much smaller than in QWs with ternary compounds, such as InGaN/GaN. However, every imperfection changes the width of the QWs (and hence, the internal electric field) and introduces strain, which causes a piezoelectric effect. These effects lead to changes in the local energy. During PL measurements, the photo-excited particles are initially randomly distributed, and then they diffuse to the local energy minima [22]. The decay times of the free particles and particles that are localized in the potential minima are different. This localization process depends on temperature, and it can contribute to an anomalous energy-temperature dependence, which is described as the ‘S-shape’.

Fairly recently, a new interesting explanation of the ‘S-shape’ behavior was proposed by Langer et al. [27]. According to their arguments, the piezoelectric field may induce a difference in the recombination rates, which have exponential energy dependence for radiative transitions, and are energy independent for the non-radiative recombination. The model describes the temperature dependence of PL and the PL lifetimes of the polar and non-polar InGaN/GaN QW structures, and it consistently explains both the blue shift within the ‘S-shape’ in polar QWs, and the lack of an ’S-shape’ in non-polar QWs, by the difference between the radiative recombination mechanisms in polar and non-polar indium-containing QW structures. Such a difference has also been observed by Badcock et al. in polar and non-polar InGaN/GaN LED structures [28]. 

The model that is proposed by Langer et al. can also be applied to account for the S-shape-like behavior that is observed in polar GaN/AlGaN structures [26], where localization is minimized (it can result from fluctuations in the QW width only), but the polarization-induced electric fields are more intense. To address this issue, a pair of polar and non-polar GaN/AlGaN multi-quantum well (MQW) samples with the same geometry were grown and analyzed in detail by time-resolved photoluminescence (TRPL). Additionally, the impact of built-in electric fields on the PL properties of such GaN/AlGaN MQWs was assessed by a comparative study of the pressure dependence of the photoluminescence (PL) of non-polar and polar structures. The built-in electric field in polar QWs leads to a decrease in the transition energy and lowers the quantum efficiency. Both of these effects are manifestations of the quantum-confined Stark effect (QCSE). Non-polar systems are free from such negative features. Moreover, for polar QWs, the observed pressure coefficients of the PL energy (*dE_PL_/dp*) are reduced relative to those of the GaN energy gap [29,30,31,32,33,34,35,36,37,38,39,40,41]. This effect is caused by the hydrostatic-pressure-induced increase in the piezoelectric field in quantum structures [33,34,35,42]. In contradiction, because of the lack of QCSE, non-polar samples are expected to follow the pressure dependence of the GaN bandgap [38].

The paper is organized as follows: In Section 2, we describe the investigated samples and the experimental methods. In Section 3, the results of the temperature-dependent PL, the TRPL, and high-pressure studies of the polar and non-polar GaN/AlGaN structures are reported and discussed. Finally, a summary and the conclusions are presented in Section 4.

## 2. Samples and Experimental Techniques

### 2.1. Sample Growth and Structural Characterization

**Figure 1:**
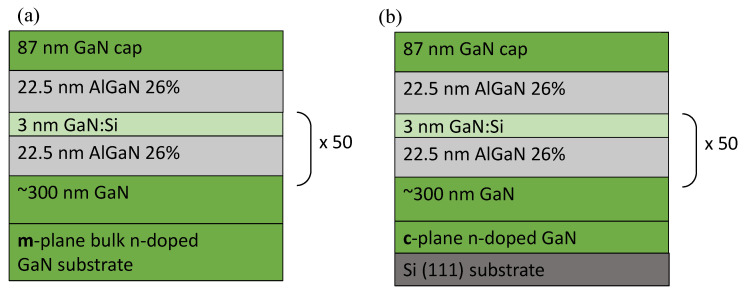
The scheme of the (**a**) non-polar and (**b**) polar GaN/AlGaN MQW structures.

A pair of polar and non-polar GaN/AlGaN MQWs with the same geometry was synthesized by plasma-assisted molecular-beam epitaxy (PAMBE). The samples were grown simultaneously on 300 nm-thick GaN buffer layers that were grown on m-plane bulk GaN and a c-plane GaN-on-Si(111) template. The growth conditions are described elsewhere [43,44]. Both samples consisted of 50 periods of 3 nm-thick GaN QWs and 22.5 nm-thick Al_0.26_Ga_0.74_N quantum barriers (QBs), which were capped by 87 nm of GaN. To populate the ground state in the conduction band, each GaN layer was doped with Si to a concentration of 2 × 10^19^ cm^−3^. The schematic drawing of the samples is presented in Figure 1.

**Figure 2:**
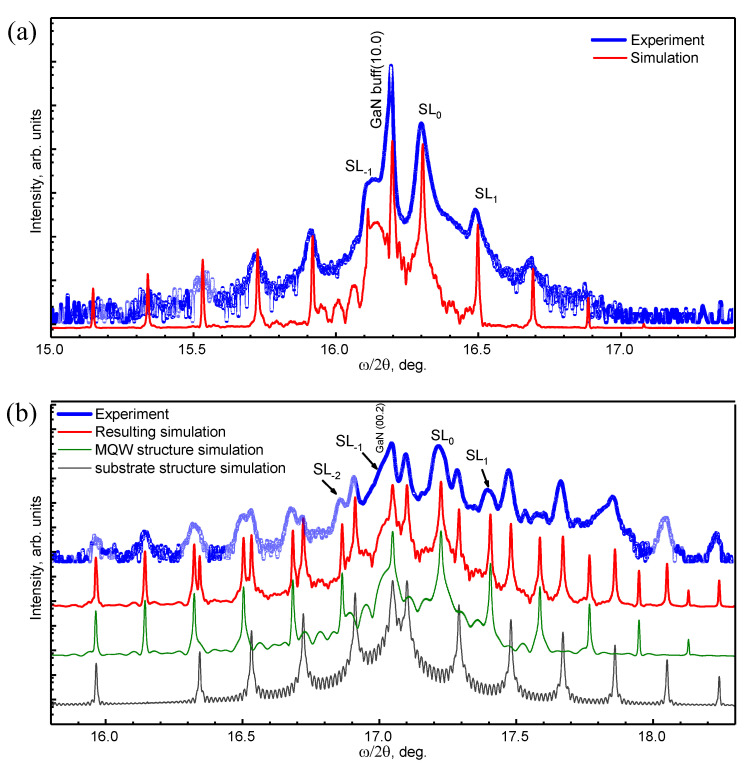
X-ray ω/2Θ scans of the (**a**) non-polar and (**b**) polar GaN/AlGaN (3 nm/22.5 nm) MQW structures.

The structural characterization was performed by high-resolution X-ray diffraction (HRXRD) and high-resolution (scanning) transmission electron microscopy [(S)TEM]. HRXRD measurements were performed with a Panalytical X’Pert Pro MRD X-ray diffractometer, operating at the Cu_Kα_1_ wavelength, and equipped with a hybrid two-bounce Ge (220) monochromator, and a threefold Ge(220) analyzer in front of a detector (proportional or Pixcel). For each sample, two types of measurements were performed: a ω/2Θ scan of the symmetrical reflection, and a reciprocal space map (RSM) of the (-1-124) asymmetrical reflection. Figure 2 shows the ω/2Θ X-ray scans around the (10-10) reflection for the non-polar MQWs, and the (0002) reflection for the polar MQWs, together with simulations using the Epitaxy software, which confirm the compliance with the nominal structure. The analysis of the polar structure is complicated because of the presence of an AlGaN/GaN superlattice inside the GaN-on-Si(111) template used for strain management.

For both samples, the lattice parameters, the residual strain, and the dislocation densities in the templates and the superlattices (SL) were determined by using the bulk lattice parameters of the GaN and AlN of Angerer et al. [45] and Moram et al. [46] (**a**_GaN_ = 3.1893 Å; **a**_AlN_ = 3.1130 Å; **c**_GaN_ = 5.1851 Å; **c**_AlN_ = 4.9816 Å). The results are summarized in Table 1. Although the dislocation density of the polar GaN template grown on the Si(111) substrate was around one order of magnitude higher than that of the non-polar GaN grown on m-plane bulk GaN, the final dislocation densities in the MQW structures were comparable. Indeed, a change in the growth direction may result in a change in the defect concentration. This effect is most probably due to the higher lattice mismatch between the GaN and AlGaN in the non-polar plane, and to the more complex growth process along the non-polar direction that is caused by the anisotropy of the surface.

**Figure 3:**
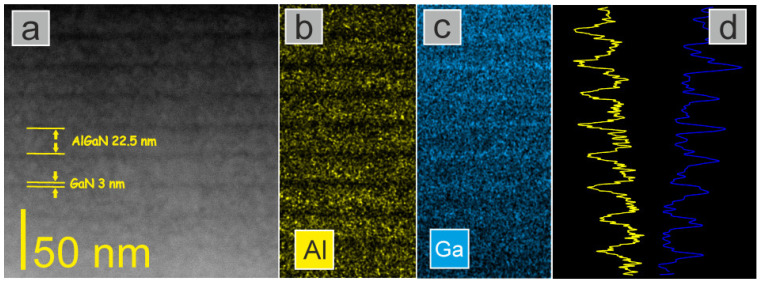
(**a**) Cross-sectional HAADF STEM image of the non-polar GaN/AlGaN MQWs, with EDS map for (**b**) Al, and (**c**) Ga distribution, and (**d**) the linear EDS profile.

The (S)TEM analysis was performed by using a Talos F200X microscope that was operated at 200 kV, and that was equipped with energy-dispersive X-ray spectrometry (EDS) and a high-angle annular dark-field (HAADF) detector. Cross-sectional TEM specimens were prepared by a standard method of mechanical pre-thinning, followed by Ar ion milling. Figure 3 presents a STEM image of the non-polar GaN/AlGaN structure with EDS maps for the Al and Ga distributions, and their linear EDS profiles, where the relationships of the Al and Ga contents are clearly visible (the gradient of contrast from the top to the bottom of the image is due to a gradient of thickness in the TEM lamela). The overall image of both samples confirms a good agreement with the designed structure.

### 2.2. Experimental Spectroscopic Techniques

PL spectra were measured at ambient and high pressure by using the deep-UV laser-lines (275.4 nm or 302.4 nm) of a continuous wave (CW) Coherent Innova 400 Ar-ion laser as an excitation source. The samples were mounted in an Oxford Optistat CF cryostat equipped with a temperature controller for low-temperature and temperature-dependent measurements. The samples were back-polished down to a thickness of 20–30 μm, and they were mounted either directly onto a sample holder for ambient-pressure experiments, or into a low-temperature diamond anvil cell (CryoDAC LT, easyLab Technologies Ltd., Almax easyLab Group Ltd., Cambridge, MA, USA) for high-pressure experiments. In both cases, the emission was dispersed by a Horiba Jobin-Yvon FHR 1000 monochromator, and the signal was detected by a liquid-nitrogen-cooled charge-coupled device camera. In the diamond anvil cell (DAC), argon was used as a pressure-transmitting medium. The samples were loaded into the cell along with a small ruby crystal. The *R_1_*-line ruby luminescence, excited by the second harmonic of an yttrium aluminum garnet laser (Nd:YAG, emission wavelength: 532 nm), was used for the pressure calibration, and the PL line-width was used to control the hydrostatic conditions in the DAC.

For the TRPL measurements, the samples were excited by the third harmonic of a pulse Ti:sapphire laser, with a selected excitation wavelength of 300 nm. The pulse frequencies were in the range of 0.2–2 MHz, with a pulse energy (*E*_p_ = 10 pJ) that corresponds to an average laser power: *P =* 2–20 μW. Additional measurements, with a pulse rate of 80 MHz, were performed with: *E*_p_ = 0.6 pJ (i.e., *P* = 50 µW). The estimated power density at the sample was in the range of 4–100 W/cm^2^. At this power, no heating effects were observed, and, thus, it could be assumed that the sample temperature was equal to the cryostat temperature of 5 K. The TRPL signal was measured by using a Hamamatsu streak camera.

## 3. Results and Discussion

### 3.1. Temperature-Dependent Photoluminescence at Ambient Pressure

**Figure 4:**
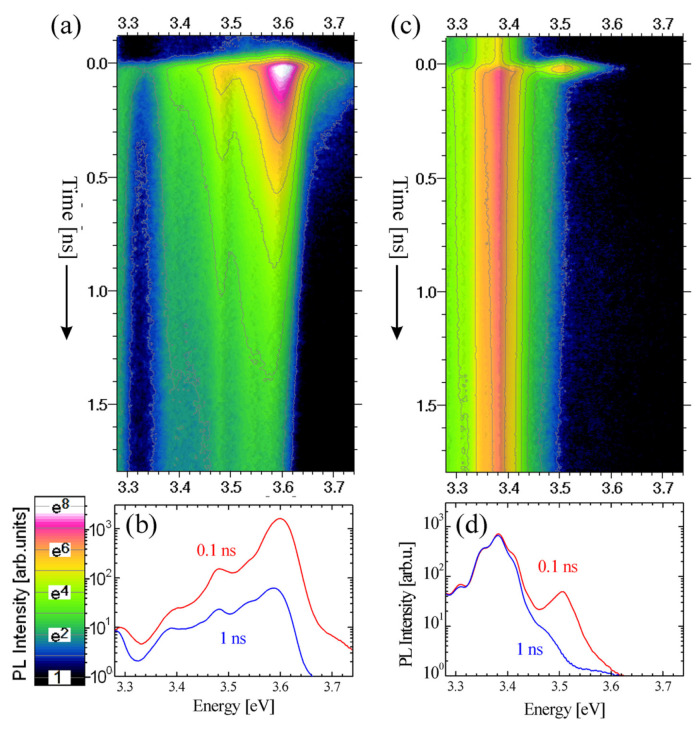
Time-resolved low-temperature PL spectra of (**a**,**b**) non-polar and (**c**,**d**) polar GaN/Al_0.26_Ga_0.74_N (3 nm/22.5 nm) MQW structures: (**a**,**c**) contour plots of PL intensity vs. time and energy; and (**b**,**d**) PL spectra at short (0.1 ns) and long (1ns) times after the laser pulse. The relatively weak lines visible around 3.5 eV originate from the GaN cap.

The presence of a built-in electric field in polar MQWs leads to a QCSE that influences both the transition energies and the recombination probabilities [47,48,49,50,51]. The electric field results in a potential gradient in the wells that separates the electrons and the holes along the **c** axis and approaches them in energy. This results in a redshift of the emission, which is approximately proportional to the electric field and the QW width, and a reduction in the overlap of the electron and the hole wavefunctions, which causes a drastic decrease in the radiative recombination rate [39,52,53]. Figure 4 shows the time evolution of the PL spectra from the (a) non-polar and (b) polar samples, and the corresponding PL spectra (Figure 4b,d) that resulted from the integration of the intensity decay over the different time intervals after the laser pulse (0.1 ns and 1 ns). The measurements were recorded at 20 K. The absence of an electric field in the non-polar sample manifests in the shorter PL peak wavelength (344.9 nm (i.e., 3.595 eV)), in comparison with its polar counterpart (366.5 nm (i.e., 3.383 eV)). These results are comparable with those reported in [38] by Teisseyre et al., where 3 nm GaN single QWs with 10 nm AlGaN QBs containing 30% of Al were investigated and were grown along the nonpolar (**a**-plane) and polar (**c**-plane) crystallographic directions. In these structures, the PL peak energies of the non-polar and polar QWs were equal to 3.608 and 3.390 eV, respectively. Furthermore, the PL decay of the non-polar sample is much faster than that of the polar structure. It should be noted that the color scale of the contour plot is logarithmic, and so the signal at the next contour is *e* times stronger (*e* = 2.718). In this situation, the exponential decay time can be read from the plot as a distance along the time axis between the contours.

In both spectra, the emission from the GaN cap is also visible. The differences in the GaN cap PL peak wavelength (of about 2 nm) may come from the different strains for the different growth directions and templates that were used to grow these structures [54,55]. Moreover, the different decay times for the GaN-related lines can be due to the different defect densities and surface effects in this topmost layer.

**Figure 5:**
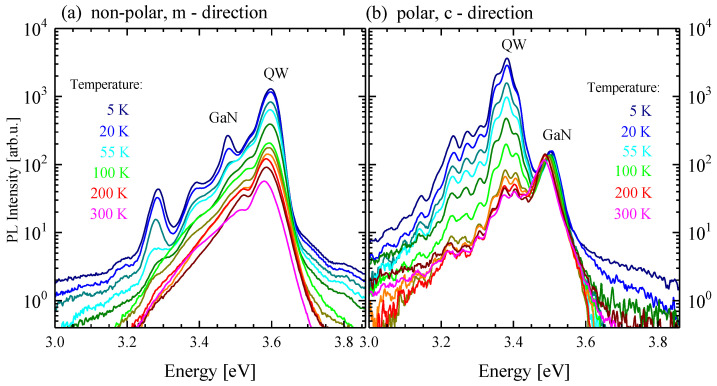
The temperature dependence of PL spectra of (**a**) non-polar and (**b**) polar GaN/Al_0.26_Ga_0.74_N (3 nm/22.5 nm) MQW structures. The oscillations visible in Panel (**b**) are due to reflection at the GaN/Si interface and the resulting Fabry–Pérot interferences.

In order to obtain a deeper insight into the recombination processes that occur in the investigated structures, measurements of PL as a function of temperature were performed. Figure 5 presents the evolution of the PL spectra of the MQWs in the temperature range from 5 to 300 K.

It is often assumed that nonradiative transitions at liquid helium temperature are negligible, and so the internal quantum efficiencies of the structures at 5 K η_int_(5 K) are assumed to be a unity [56,57], and, therefore, by comparing the ratios of the integrated intensities of both samples at 300 and 5 K, their room-temperature quantum efficiencies can be estimated. They are equal to 7.3% and 0.1% for the non-polar and polar GaN/Al_0.26_Ga_0.74_N MQW structures, respectively. This indicates a significantly stronger influence of non-radiative processes on the polar structures.

**Figure 6:**
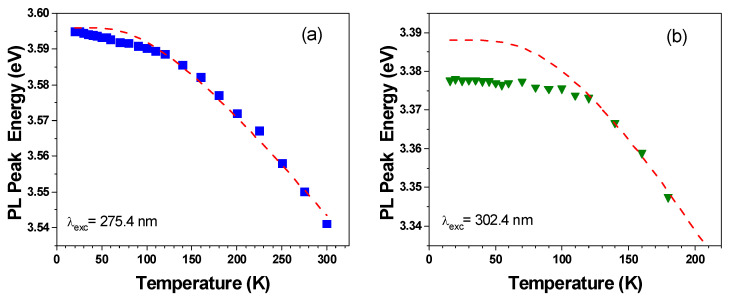
Temperature dependence of the PL peak energies of (**a**) non-polar and (**b**) polar GaN/Al_0.26_Ga_0.74_N (3 nm/22.5 nm) MQW structures. Dashed lines are fits to Equation (1) with the parameters listed in Table 2.

Figure 6 depicts the evolution of the PL peak energies of non-polar and polar GaN/AlGaN MQWs as a function of temperature, measured with a CW excitation. Similar to the InGaN/GaN structures analyzed by Langer et al. [27], the non-polar and polar GaN/AlGaN samples exhibit different temperature behavior. This is evident by comparison with the Viña model that is plotted in Figure 6, and that is based on Bose–Einstein statistics [16]:

(1) $$E\left(T\right)=E_{0}-\frac{2\alpha }{\exp(\Theta /T)-1}$$

where Θ is related to the average phonon frequency, and *α* describes the strength of the interaction. The parameters of the simulation curves are listed in Table 2. The values of *E*_0_ were adjusted to the experimental data (Θ), and the *α* for the non-polar structure was obtained by fitting the parameters to the experimental data, and for the polar structures, it was chosen to obtain the best fit to the experimental data in the high-temperature range. They are in good agreement with the data that is reported in the literature for GaN layers [58,59,60,61] that was collected by Kudrawiec et al. in [62].

The temperature dependence of the non-polar structure (Figure 6a) follows almost perfectly the expected redshift with the increasing temperature. Small discrepancies (<3 meV) can be caused by localization effects at the QW width fluctuations. Much larger deviations, reaching 10 meV, are observed for the polar MQWs (Figure 6b), up to 120 K. This dependence does not reveal a strong S-shape behavior, but it deviates significantly from the expected trend, and resembles the effect that is reported by Friel et al. for 1 nm polar GaN/Al_0.2_Ga_0.8_N MQWs [26]. This behavior cannot be explained by using Chichibu’s model of carrier localization in the local potential minima and the Boltzmann occupation of localized states because the QWs are made of binary GaN (i.e., there are no alloy fluctuations within the wells). However, such an effect could be induced by QW width fluctuations and/or alloy fluctuations in the QBs, which lead to an inhomogeneous internal electric field in the QW. Additionally, a part of the ‘S-shape’-like behavior can be due to the competition between the radiative and nonradiative recombination in a spectrum where the radiative recombination rate is wavelength-dependent.

### 3.2. Energy Dependence of the PL Decay Times

As is mentioned above, the QCSE causes a separation of the electron–hole wave functions, which results in the strong decrease in the recombination rates in polar structures. Therefore, the decay kinetics are longer in polar QWs with respect to their non-polar analogues. Furthermore, in the case of QW width fluctuations that are manifested by PL peak broadening or a multi-peak structure, the decay times that correspond to the lower-energy regions would be longer in polar QWs, and energy-independent in non-polar QWs. As the overlap matrix element in polar QWs is reduced slightly more strongly than exponentially with the QW thickness, the inhomogeneously broadened QW emission spectrum is expected to be governed by an approximately exponential energy dependence of the radiative lifetime [39,52,53]. 

**Figure 7:**
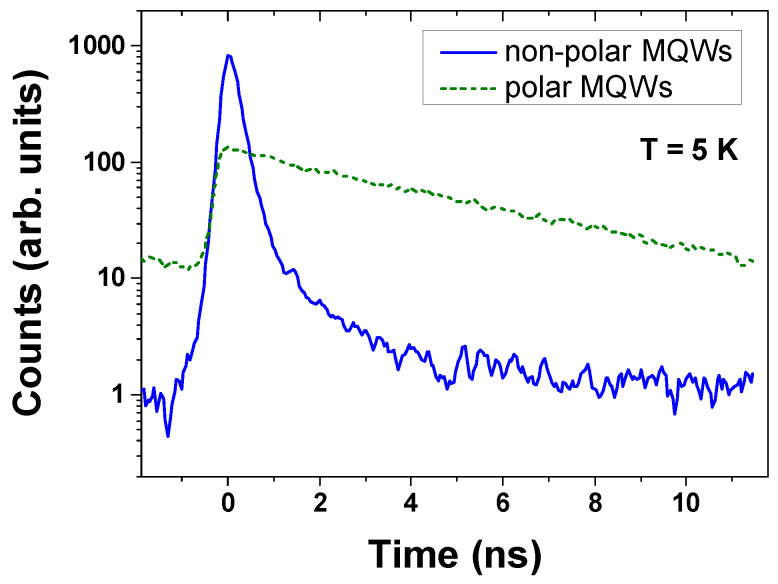
Low-temperature PL decay of non-polar (solid blue line) and polar (dashed green line) GaN/Al_0.26_Ga_0.74_N (3 nm/22.5 nm) MQW structures, measured at pulsed 300 nm-wavelength excitations.

Figure 7 presents the examples of the PL decays of non-polar and polar MQW structures measured at 5 K, which illustrate the drastic difference in the decay kinetics of these structures. The transient of the polar sample emission shows a standard, almost mono-exponential, decay, whereas the non-polar sample presents an initial fast exponential decay, and then a slower nonexponential tail. Similar decay behavior is often observed in nitride-based QWs [27]. The non-exponential tail is probably due to potential fluctuations that trap carriers and cause slower recombination, but this effect does not change the main conclusions. The initial decay is more than an order of magnitude stronger than the nonexponential tail (note the exponential scale of the vertical axis in Figure 7), and so, in our analysis, we took into account only the first exponential part. The decay in the non-polar structure is very fast compared to the polar sample because the electrons and holes are not separated by the electric field, and the overlap of the electron and hole wave functions is high, which results in a high radiative recombination rate. It should also be noted that just after excitation, the non-polar sample has a higher intensity, which indicates a more efficient radiative recombination.

**Figure 8:**
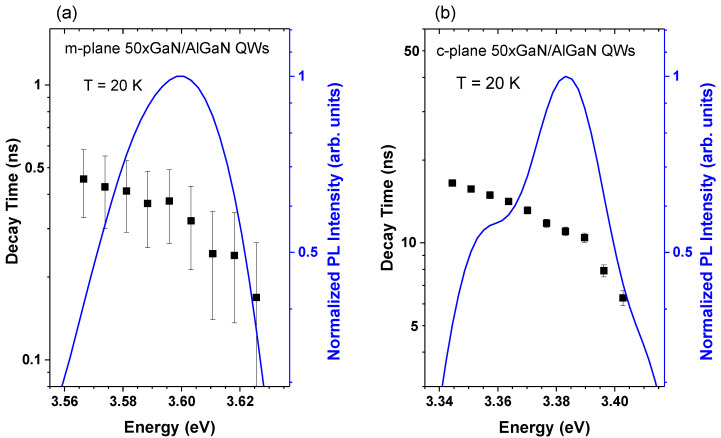
Low-temperature PL decay times determined for different spectral positions for the non-polar (**a**) and polar (**b**) GaN/AlGaN MQWs. The normalized low-temperature time-integrated PL spectra are shown as blue solid lines. The relative changes in the decay times are the same for a better comparison.

In order to directly compare our TRPL data with the results of Langer et al. [27], the decay times were determined in the same way (i.e., by an exponential fit of the initial part of the intensity decay, where the intensity drops from its maximum value to 1e (approximately 60%) of the maximum. Figure 8 shows the low-temperature PL decay times that were established for different spectral positions for the non-polar (m-plane) and polar GaN/AlGaN MQWs, superimposed on the time-integrated PL spectra. The decay times in the non-polar structure are in the range of 0.17 ÷ 0.45 ns, which corresponds to a high overlap of the electron and hole wave functions. These values are in accordance with the decay times that were determined in the same way by Langer et al. [27] (0.2 ÷ 0.3 ns). The polar MQWs exhibit much longer PL decay times, of the order of several nanoseconds. They are slightly longer than those reported by Langer et al. [27] for InGaN/GaN QWs, which reflects the differences in both the QW widths and the polarization fields in the structures.

In contrast to Langer’s results, the decay times of the non-polar structure are not energy-independent but display a nearly exponential energy dependence, with a slope that is similar to that observed in the polar sample, even though the time range differs by almost two orders of magnitude. This effect may be due to the slower recombination of carriers trapped in the local potential minima, which is the result of QW width fluctuations. Furthermore, the decay time of the polar MQWs drops fast in the emission range of 3.39–3.40 eV, which can be caused by the overlap of the MQW emission with the emission from the GaN cap, with much shorter decay times, due to the negligible QCSE (see Figure 4). In further analysis, we will focus on the results in the part of the spectra with an intensity exceeding half of the maximum intensity.

Following the analytical model that was developed by Langer et al., we can estimate the value of the total amount of the blue shift Δ*E* because of the energy dependence of the decay time that is caused by the presence of an internal electric field, which is expressed by the formula [27]:

(2) $$\Delta E=s_{\Gamma }\cdot \sigma^{2}$$

where *s*_Γ_ is a parameter describing the steepness of the slope in a logarithmic plot of relative decay times in a function of energy in the eV^−1^ units, and *σ* is the inhomogeneous broadening parameter of a Gaussian function. In the case of the analyzed GaN/AlGaN structures, this “blue shift” Δ*E* would contribute to the difference between the low-temperature bandgap energy predicted by the Viña model, and the PL energy observed experimentally (see Figure 6).

The *s*_Γ_ parameter can be determined for the polar structure straight from the data that are presented in Figure 8b. For the decay time that corresponds to the central part of the PL spectrum, it is equal to about (13.0 ± 0.3) eV^−1^. Assuming a Gaussian shape of the PL spectra, and taking into account that the low-temperature full width at half maximum of both the non-polar and polar samples was equal to about 50 meV, this gives: *σ* ≈ (22 ± 1) meV. Thus, the resulting estimate of the blue shift of the PL energy is due to the energy dependence of the decay times: *ΔE* ≈ (6.3 ± 0.7) meV. This value is smaller than that evaluated from the data presented in Figure 6b. Additionally, as shown in Figure 8, the slope of the energy dependence of the decay times of non-polar and polar structures is similar in both cases, and the linewidths are similar too. These results indicate that the competition between radiative and non-radiative recombination with different energy dependences, which is the basis of Langer’s model, is not the dominant effect that is responsible for the ‘S-shape’ behavior of PL energy, and the localization effects due to the QW width fluctuation are important in the GaN/AlGaN system.

### 3.3. Temperature Dependence of PL Decay Times

**Figure 9:**
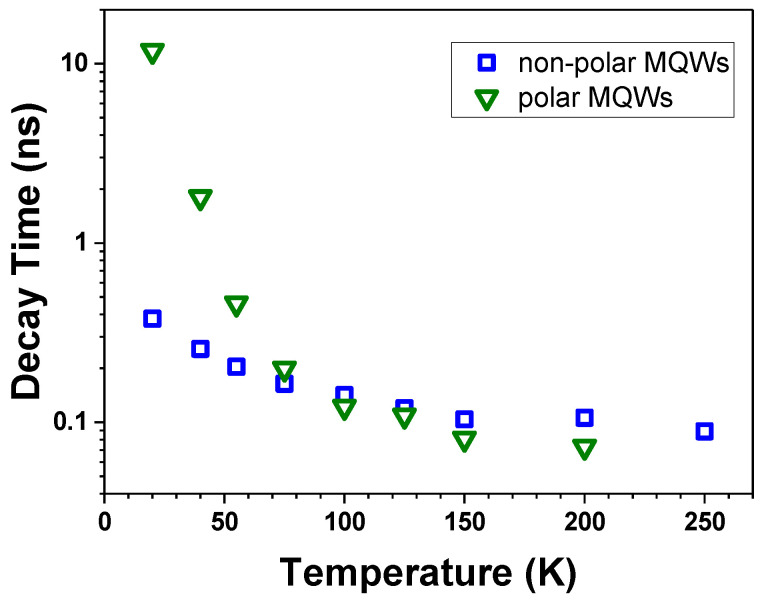
Temperature dependences of PL decay times of the non-polar (blue squares) and polar (green triangles) GaN/AlGaN MQWs, measured at the maximum of the PL.

Figure 9 presents the evolution with the temperature of the PL decay times of non-polar and polar GaN/AlGaN MQWs at the maximum of the PL. The decay time in non-polar QWs decreases by approximately a factor of 4 when the temperature increases from 20 K to 200 K, whereas the corresponding decrease in the decay time in polar QWs exceeds a factor of 100. It is worth noting that the strongest drop occurs in the range of 20–100 K. This effect can be attributed to the thermal activation of non-radiative recombination, which is especially pronounced in the polar structure because of the longer radiative decay time. At temperatures exceeding 100 K, where the recombination on the defects dominates, the decay times of the non-polar and polar structures became similar. This result confirms the findings of the XRD analysis, which revealed comparable defect densities in both structures (see Table 1); however, it should be kept in mind that the resolution limit of our system is around 0.1 ns. Note that the reduction in the decay time cannot be due to the thermal occupation of higher potential states, since this would lead to a blue shift of the PL energy, which was not observed in our experiments.

The significantly stronger influence of non-radiative processes in polar structures also manifests by the much stronger thermal quenching of the luminescence of these structures in comparison to their non-polar analogues. This can be caused by the generally lower radiative emission rate of polar QWs, which is due to a lower overlap of the electron and hole wave functions, which results in the overall greater influence of non-radiative transitions on the deexcitation processes in polar QWs.

### 3.4. Pressure Dependence of the Luminescence Spectra at Low Temperature

**Figure 10:**
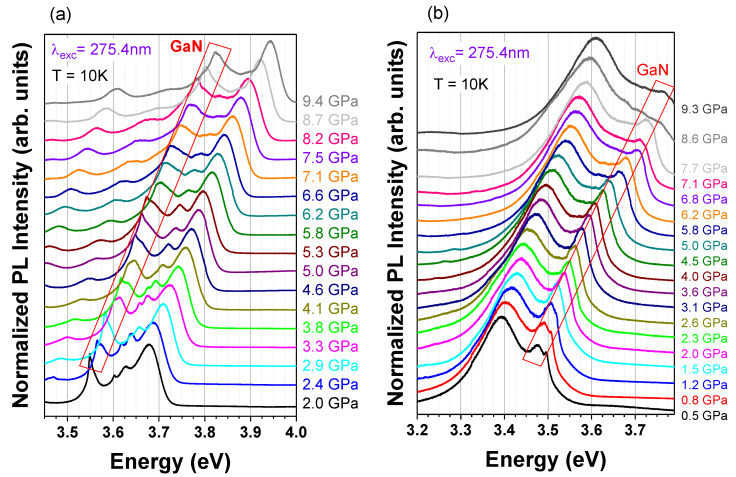
Pressure dependence of the low-temperature PL spectra of a non-polar (**a**) and polar (**b**) GaN/AlGaN MQWs. The emission from the GaN cap (marked by red tetragons) is visible in the whole range of pressures.

**Figure 11:**
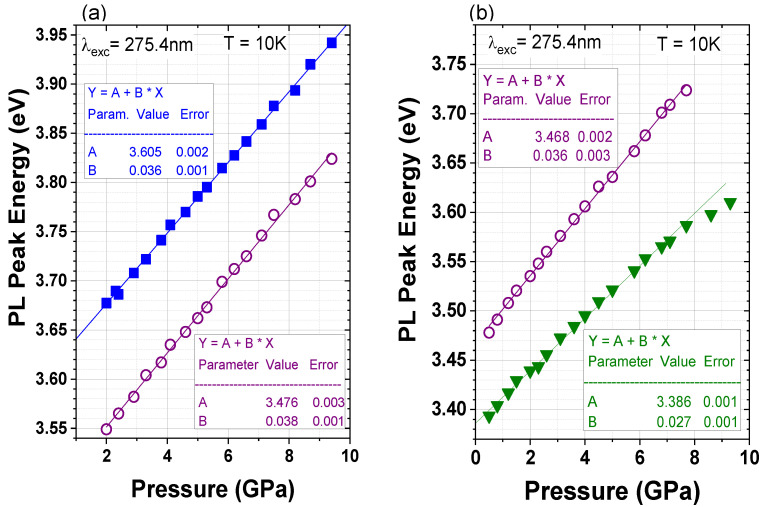
PL peak energies as a function of pressure for non-polar (**a**) and polar (**b**) GaN/AlGaN MQWs (filled symbols) and the GaN cap (open symbols). The pressure coefficients (*dE_PL_/dp*) are determined as a slope of the linear fits to the experimental data.

The comparative study of non-polar and polar GaN/AlGaN MQWs also included high-pressure measurements. The results are shown in Figure 10, and the spectral peak positions of the PL of the MQWs, and the GaN caps as a function of pressure, are presented in Figure 11.

The experiment revealed a generally linear dependence of the PL peak energy on the applied pressure. Some deviation from the linearity, which is visible in the polar sample at the highest pressure, can be associated with the presence of deep localized states, which can become involved in the radiative recombination process at a sufficiently high bandgap energy, the effect of which is reported in the literature [36,39]. These deviated data points have been excluded from the further analysis of the pressure behavior of MQWs. 

The slope of a linear fit to the data gives pressure coefficients that are equal to 36 ± 1 meV/GPa for the non-polar MQWs, and to 27 ± 1 meV/GPa for the polar MQWs. As expected, the non-polar structures revealed almost the same pressure behavior as the GaN cap. The value is consistent with previous reports for thick GaN layers or bulk GaN, where pressure coefficients around 40 meV/GPa were obtained [55,63,64]. Slight differences can be caused by different strains in both structures (see Table 1).

The pressure coefficient of the polar structure (Figure 11b) is close to the value of 30 meV/GPa that was obtained from the DFT calculations for the 3 nm-wide n-doped MQWs with the same charge density of 2 × 10^19^ cm^−3^, but narrower 4 nm Al_0.25_Ga_0.75_N QBs [41]. These results are also in good accordance with the pressure behavior of 3 nm GaN single QWs with AlGaN QBs containing 30% of Al, which is reported by Teisseyre et al. in [38]. In these structures, the PL pressure coefficients of the non-polar and polar QWs were equal to 42 ± 1 meV/GPa and 21 ± 1 meV/GPa, respectively. Some differences in the PL pressure coefficients indicate that, in the reported case, the QW width could differ by ±1 monolayer than the assumed QW width of 3 nm. The reduction in the pressure coefficients of the polar structures with respect to those of the bulk GaN was observed before and has been explained by the hydrostatic-pressure-induced increase in the piezoelectric field in quantum structures and by the non-linear behavior of the piezoelectric constants [36,38,41].

## 4. Conclusions

Polar and non-polar GaN/AlGaN MQW structures, with the same geometry and composition, were synthesized by PAMBE. Their structures and quality were validated by XRD and TEM characterization, and it was shown that, except for the polarity, both samples had similar structural properties and dislocation densities. However, their optical properties were very different. In comparison to non-polar structures, the built-in electric field in polar MQWs induces a redshift of the PL emission, a stronger temperature quenching of the PL, and a drastically lower radiative recombination rate. Moreover, the temperature dependence of the PL energy in these structures was different, being almost perfectly consistent with the Viña model, which is based on the Bose–Einstein statistic for the non-polar structure, and showing clear discrepancies at temperatures below 120 K for its polar counterpart. This discrepancy cannot be explained by using the model that is based on different energy dependencies of the radiative and non-radiative recombination rates that is described by Langer et al. in [27], which was applied successfully to InGaN/GaN structures. Our results show that the localization effects caused by the QW width fluctuations or alloy inhomogeneities in the QBs, accompanied by the intense internal electric field, have a profound effect on the emission properties of polar structures. This is generally the origin of the ‘S-shape’-like behavior in polar AlGaN/GaN QWs.

High-pressure studies of GaN/AlGaN MQWs revealed that non-polar QWs have a pressure coefficient of PL energy that is similar to the pressure coefficient of bulk GaN, while the pressure coefficient of polar QWs is highly reduced with respect to that of the bulk GaN. This effect has been observed in other nitride structures and is ascribed to the hydrostatic-pressure-induced increase in the piezoelectric field in quantum structures, and to the non-linear behavior of the piezoelectric constant. The obtained results show that the growth direction constitutes a very important factor in determining the basic optical properties of nitride heterostructures.

## Figures and Tables

**Table 1:** Structural characteristics of the non-polar and polar GaN/AlGaN structures on GaN substrates: lattice parameters, in-plane strains, and dislocation densities of the GaN templates and the SLs. Strains in GaN QWs and AlGaN QBs ($\varepsilon_{c}$ and $\varepsilon_{a}$) are determined comparatively to bulk GaN, or a hypothetical AlGaN layer in SL, respectively. Strains in SLs ($\varepsilon_{c}^{SL}$ and $\;\varepsilon_{a}^{SL}$) are determined comparatively to substrate.

MQWs Type	Layer		c (Å)	a (Å)	$\varepsilon_{c}$ (%)	$\varepsilon_{a}$ (%)	$\varepsilon_{c}^{SL}$ (%)	$\;\varepsilon_{a}^{SL}$	Dislocation Density (10^9^ cm^−2^)
Non-polar structure	GaN template		5.1944	3.1888	0.179	−0.016	-	-	0.11
	SL	GaN	5.1389	3.1685	−0.899	-	−1.068	-	1.76
		AlGaN			0.329	−		-	
Polar structure	GaN template		5.1859	3.1866	0.0154	−0.085	-	-	1.38
	SL	GaN	5.1363	3.1805	-	−0.277	-	−0.191	1.63
		AlGaN			-	0.299	-		

**Table 2:** Simulation parameters of the Bose–Einstein expression for the temperature dependence of PL peak energies of non-polar and polar MQWs.

MQWs Type	*E*_0_ (eV)	*α* (meV)	Θ (K)
Non-polar	3.596	52 ± 8	326 ± 29
Polar	3.388	100	326

## Data Availability

The data underlying this article will be shared upon reasonable request from the corresponding author.
